# Network Slicing in 6G: A Strategic Framework for IoT in Smart Cities

**DOI:** 10.3390/s24134254

**Published:** 2024-06-30

**Authors:** Ahmed M. Alwakeel, Abdulrahman K. Alnaim

**Affiliations:** 1 Faculty of Computers & Information Technology, University of Tabuk, Tabuk 71491, Saudi Arabia; aalwakeel@ut.edu.sa; 2 College of Business Administration, King Faisal University, Al Ahsa 31982, Saudi Arabia

**Keywords:** 6G, slicing, IoT, smart cities, cloud computing

## Abstract

The emergence of 6G communication technologies brings both opportunities and challenges for the Internet of Things (IoT) in smart cities. In this paper, we introduce an advanced network slicing framework designed to meet the complex demands of 6G smart cities’ IoT deployments. The framework development follows a detailed methodology that encompasses requirement analysis, metric formulation, constraint specification, objective setting, mathematical modeling, configuration optimization, performance evaluation, parameter tuning, and validation of the final design. Our evaluations demonstrate the framework’s high efficiency, evidenced by low round-trip time (RTT), minimal packet loss, increased availability, and enhanced throughput. Notably, the framework scales effectively, managing multiple connections simultaneously without compromising resource efficiency. Enhanced security is achieved through robust features such as 256-bit encryption and a high rate of authentication success. The discussion elaborates on these findings, underscoring the framework’s impressive performance, scalability, and security capabilities.

## 1. Introduction

We expect that the 6G communication era will begin in 2030. This sixth-generation wireless technology will definitely have a great impact on our daily lives [1]. It has a capability that is a very huge revolution. The 6G technology is the main key of the wireless era. It will excel in all the features and drawbacks of the previous G wireless technology, in which it surpasses 5G, 4G, and 3G [2]. Once more, we are waiting for a massive information and communication revolution with technology upgrading technologies. For instance, the integration of the Internet of Things in smart cities can be seen as a driving force to this change. It is critically important to combine digital intelligence with urban infrastructure in order to improve efficiency, sustainability, and quality of life. The unique concept of network slicing is central to this strategy, as it fundamentally transforms the deployment and utilization of networks. Inside the realm of 6G, network slicing facilitates the development of personalized, virtualized networks designed to meet the unique requirements of various applications inside smart urban environments [3]. This offers not only unparalleled levels of connectivity but also the versatility to adjust to the ever-changing and diverse communication needs of IoT devices spread throughout metropolitan environments [4]. Smart cities exemplify the intricacy of contemporary urban life through their sophisticated network of networked gadgets, sensors, and systems [5]. The combination of 6G with network slicing is a powerful force that has the potential to revolutionize communication infrastructures by optimizing them in ways that were previously seen as futuristic [6]. The incorporation of 6G and network slicing offers the potential for a communication framework that is not only efficient and resilient but also capable of scaling and adapting to the changing requirements of urban areas. Our goal is to contribute to the realization of smart cities by addressing the intricate relationship between 6G and network slicing. We seek to provide seamless connectivity, intelligent resource allocation, and dynamic flexibility, which are essential for creating a sustainable and connected urban future [7]. 

The combination of 6G with network slicing in the context of smart cities presents uncharted issues, necessitating a thorough examination [8]. The effective allocation of resources and suitable configurations for dividing tasks are crucial for satisfying the ever-changing communication requirements of various Internet of Things (IoT) applications [9]. Given the large volume of sensitive data generated by smart city IoT, it is crucial to implement strong cryptographic techniques to effectively solve security and privacy issues. To overcome the interoperability issues between various network slices and devices, it is crucial to address the heterogeneous character of IoT ecosystems. It is essential to create adaptable and long-lasting plans for incorporating network slicing into 6G in order to handle the expected increase in smart city IoT applications [10].

The swift advancement of communication technologies, specifically the shift from 5G to 6G, offers an unparalleled chance to include the Internet of Things (IoT) in smart cities. Network slicing is a crucial factor in facilitating this integration. It is a groundbreaking technique that enables the formation of virtualized, autonomous, and customized network instances designed for specific use cases [10]. The potential of network slicing to improve the effectiveness and adaptability of communication networks has been demonstrated. However, its implementation and optimization for the specific needs of smart cities’ IoT in the context of 6G have not been investigated. This research aims to address the following concerns:

- Identifying the most efficient configurations for network slices within the context of 6G to meet the diverse communication requirements of smart cities’ IoT applications.
- Addressing the challenge of resource allocation across network slices to ensure seamless connectivity and fast communication for the multitude of IoT devices in smart city environments.
- Examining and proposing robust security measures to protect data transmitted within network slices, considering the privacy concerns associated with the vast amount of sensitive information generated by smart city IoT devices.
- Investigating potential challenges in integrating different network slices and devices, aiming to develop guidelines that facilitate smooth communication across diverse IoT ecosystems within smart cities.
- Developing scalable implementation strategies for network slicing technologies in 6G, considering the anticipated expansion of smart city IoT applications and the need for adaptable and future-proof communication infrastructure.

The main contributions of this study are as follows:

- Comprehensive setup examination: conducting an exhaustive exploration of innovative techniques to dynamically ascertain optimal setups for network slices in 6G, specifically tailored for smart cities’ IoT applications.
- Resource allocation strategies: introducing novel strategies to overcome resource allocation challenges, ensuring efficient and minimal-delay connectivity for a wide range of IoT devices in smart urban environments.
- Enhanced data security: improving the security of sensitive data transmitted within network slices for smart city IoT applications by implementing novel security techniques such as encryption and authentication protocols.
- Interoperability solutions: addressing interoperability obstacles by developing cutting-edge communication protocols and standards to enable seamless communication between different network slices and IoT devices.
- Scalable implementation frameworks: introducing scalable and future-proof strategies for implementing network slicing technologies in 6G, providing a robust foundation for accommodating the anticipated expansion of smart city IoT applications.
- This research endeavors to advance our understanding of the implications, challenges, and solutions related to leveraging network slicing technologies in 6G for integrating the IoT in smart cities.

This paper is organized into five main sections. The introduction offers a comprehensive review of 6G and network slicing technologies within the framework of smart cities’ IoT. A thorough literature review rigorously assesses previous research, highlighting any areas that have not been adequately explored and situating the current study within the wider academic conversation. The methodology section delineates the specific methods utilized to investigate and exploit network slicing in the context of 6G. The results and discussion entail the presentation of empirical results and the subsequent interpretation of those findings. The conclusions provide a concise and comprehensive overview of the main findings, contributions, and possible directions for future research, presenting a coherent and perceptive examination of the overlap between network slicing and smart cities’ IoT in the 6G age.

## 2. Literature Review

The emergence of 6G technology is anticipated to revolutionize various sectors, including smart cities [11,12,13,14,15,16], through enhanced connectivity [17,18,19,20,21], efficiency [3,22,23,24,25], and security [13,24,25,26]. This section reviews related work in the context of 6G and its applications, particularly focusing on network slicing, IoT integration, and the challenges faced in smart city deployments.

Shen et al. discussed the research challenges and opportunities associated with 6G technology, emphasizing its transformative potential across multiple domains, including smart cities. They highlighted key areas requiring further investigation, such as spectrum management and AI integration [1]. Nawaz et al. provided an extensive survey on quantum machine learning for 6G communication networks, outlining the state-of-the-art techniques and future directions for enhancing 6G capabilities [2].

Network slicing is a pivotal concept in 6G, enabling the creation of multiple virtual networks on shared physical infrastructure. Zhou et al. explored the automatic network slicing for IoT in smart cities, proposing a framework that ensures efficient resource allocation and improved performance [4]. Nguyen et al. conducted a comprehensive survey on the 6G Internet of Things (IoT), addressing the integration of network slicing and highlighting the need for robust security measures to handle the vast amount of data generated by IoT devices in smart cities [5].

Security and privacy are critical concerns in 6G deployments. Moya Osorio et al. discussed the security and privacy issues in 6G-enabled Internet of Vehicles (IoV), providing insights into potential solutions and challenges [13]. Andronie et al. focused on big data management algorithms and deep learning-based object detection technologies, emphasizing the importance of secure data handling in smart city applications [16].

Puspitasari et al. examined emerging technologies for 6G communication networks, particularly the application of machine learning approaches to enhance network efficiency and performance [6]. Dogra et al. surveyed beyond 5G networks, highlighting the role of AI in optimizing 6G network architecture and addressing emerging technological challenges [19].

Scalability is essential for the successful implementation of 6G in smart cities. Mahmood et al. identified key drivers and enablers for machine-type communications in the 6G era, emphasizing the need for scalable network solutions [8]. Kumar et al. explored the potential of 6G for promoting sustainability, discussing how 6G can contribute to greener future technologies [14].

Several comprehensive surveys and reviews provide a broad understanding of 6G and its applications. Murroni et al. presented a survey on 6G technologies, emphasizing their role in smart city development and outlining the challenges and opportunities ahead [7]. Guo et al. conducted a thorough survey on enabling massive IoT toward 6G, highlighting the technical requirements and potential applications [21].

The integration of advanced technologies such as blockchain and AI is pivotal for the evolution of 6G. Fadhil proposed a framework integrating 6G communication and blockchain technology to enhance smart city advancements, focusing on secure and transparent communication [3]. Al Amin et al. discussed the convergence of AI and Mobile Edge Computing (MEC) for autonomous IoT service provisioning in beyond 5G networks, illustrating the potential benefits of this integration for 6G [18].

These studies collectively underscore the transformative potential of 6G technologies in smart cities, particularly through the innovative application of network slicing techniques, enhanced security measures, and the integration of AI and blockchain technologies. The research also highlights the need for scalable, efficient, and sustainable network solutions to meet the diverse and dynamic requirements of smart city IoT applications.

Table 1 shows the comparative analysis of previous state-of-the-art studies.

## 3. Materials and Methods

This section provides a detailed explanation of the methods used in our research project. Our focus is on developing and implementing network slicing technologies for smart cities’ IoT inside the framework of 6G. Our research aims to utilize network slicing to effectively address the varied and ever-changing communication requirements of IoT applications in smart cities. Our research takes a practical approach, seeking to connect the theoretical knowledge of network slicing technologies in 6G with real-world applications designed specifically for the distinct needs of smart cities’ IoT. Our aim is to explore the complexities of network slicing in order to create and enhance slicing configurations that effectively distribute resources, guaranteeing uninterrupted connectivity, fast communication, and optimal performance for the numerous IoT devices located throughout smart city environments.

### 3.1. Designing Network Slicing for 6G

**Figure 1:**
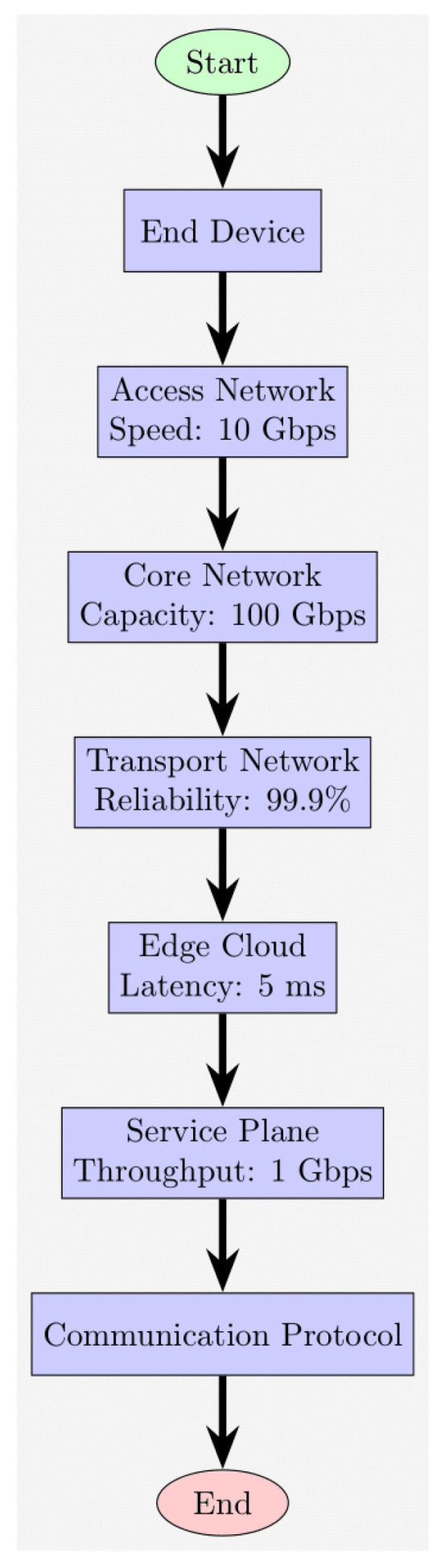
Sixth-generation network slicing flow process.

In order to implement the design of network slicing technologies for 6G within the framework of smart cities’ IoT, we develop a comprehensive mathematical model that encompasses the essential characteristics, limitations, and goals of our practical research. As illustrated in Figure 1, the architecture of the 6G network slicing comprises several unique components, namely the Access Network, Core Network, Edge Cloud, Transport Network, and Service Plane. This architectural design facilitates the effective allocation and segregation of resources for a wide range of services. Our mathematical model aims to precisely quantify the intricate interactions and resource allocations within this network slicing architecture. Leveraging mathematical formalisms, we articulate the dynamics of resource provisioning, service prioritization, and performance optimization. By formulating mathematical equations that capture the relationships between network components, traffic patterns, and service requirements, our model provides a rigorous framework for analyzing and optimizing network slicing configurations. Through mathematical modeling, we can systematically evaluate the performance implications of various network slicing configurations under different scenarios. This enables us to identify optimal resource allocations, mitigate performance bottlenecks, and enhance the overall efficiency and effectiveness of 6G network slicing in smart cities’ IoT environments.

#### 3.1.1. Mathematical Model

Let X represent the decision variables for the optimal slicing configuration. Our objective is to minimize the overall cost function f(X) subject to certain constraints. The mathematical formulation of the optimization problem is as follows:

(1) $$\begin{array}{llll} & \underset{X}{minimize} & & f(X)\\ & subjectto & & g_{i}(X)\leq 0,\;\;\;\;i=1,\ldots ,m\\ & & & h_{j}(X)=0,\;\;\;\;j=1,\ldots ,p, \end{array}$$

where f(X) represents the objective function, and $g_{i}(X)$ and $h_{j}(X)$ are inequality and equality constraints, respectively.

#### 3.1.2. Objective Function

The objective function f(X) is defined as a weighted sum of various performance metrics, capturing the essence of the optimal slicing configuration:

(2) $$f(X)=\sum_{k=1}^{N}w_{k}\cdot \left[\alpha_{k}\cdot Metric_{k}(X)+\beta_{k}\cdot {\left(\frac{\gamma_{k}\cdot Constraint_{k}(X)}{\delta_{k}+Constraint_{k}(X)}\right)}^{\theta_{k}}\right],$$

where $Metric_{k}(X)$ represents the performance metric for the k-th aspect of the optimal slicing configuration, and $w_{k}$ is the weight assigned to the k-th metric.

**Figure 2:**
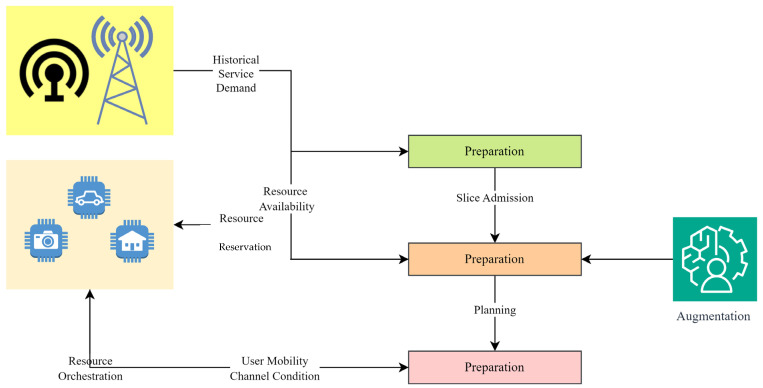
Designing network slicing for 6G.

In Figure 2, an illustration of the network slicing design process for 6G is provided. The process entails collecting specifications, delineating measurements, recognizing limitations, setting goals, constructing a mathematical framework, optimizing the arrangement, assessing effectiveness, fine-tuning variables, and concluding the blueprint.

Our objective is to use a mathematical framework to define our research topic and create network slices that efficiently allocate resources, provide fast communication, and fulfill the varied and ever-changing communication requirements of smart cities’ IoT applications in the context of 6G.

### 3.2. Implementation of Smart Cities’ IoT in Context of Network Slicing Technologies in 6G

In this section, we delve into the concrete deployment of network slicing technologies tailored for smart cities’ IoT within the framework of 6G. Our approach revolves around the development of a comprehensive mathematical model, which serves as the cornerstone for guiding the implementation process. The essence of our mathematical model lies in its ability to formulate essential equations that govern various aspects of the implementation strategy. By encapsulating critical parameters, constraints, and objectives, these equations provide actionable insights into resource allocation, service provisioning, and performance optimization. Through rigorous mathematical formalisms, we establish a systematic framework for orchestrating the deployment of network slicing technologies in smart cities’ IoT environments. This framework enables us to precisely define deployment strategies, allocate resources judiciously, and optimize network configurations to meet the diverse communication requirements of 6G-enabled IoT applications in smart urban settings. By leveraging mathematical modeling, we can navigate the complexities inherent in deploying network slicing technologies within the dynamic and heterogeneous landscape of smart cities. Our approach empowers stakeholders to make informed decisions, mitigate deployment risks, and maximize the utility of 6G network slicing for advancing the goals of smart cities’ IoT.

#### 3.2.1. Mathematical Model

In order to optimize the implementation of network slicing for smart cities’ IoT in the 6G era, we develop a mathematical model that encompasses the crucial factors and concerns. The model is specifically developed to enhance the configuration by utilizing predetermined metrics and objectives.

##### Objective Function

The overarching objective is formulated as follows:

(3) $$f(X)=\sum_{k=1}^{N}w_{k}\cdot Metric_{k}(X),$$

where the following terms are used:

- X represents the decision variables for the optimal slicing configuration.
- N is the number of metrics considered.
- $Metric_{k}(X)$ denotes the performance metric for the k-th aspect of the optimal slicing configuration.
- $w_{k}$ is the weight assigned to the k-th metric.

##### Constraints

The optimization problem is subject to various constraints, ensuring the feasibility of the network slicing configuration. The general form of the constraints is given by the following:

(4) $$g_{i}(X)\leq 0,\;\;\;\;i=1,\ldots ,m,$$

(5) $$h_{j}(X)=0,\;\;\;\;j=1,\ldots ,p,$$

where the following terms are used:

- $g_{i}(X)$ and $h_{j}(X)$ are inequality and equality constraints, respectively.
- m is the number of inequality constraints, and p is the number of equality constraints.

##### Decision Variables

The decision variables X denote the parameters that establish the network slicing configuration. These factors are fine-tuned to attain the intended level of performance. The decision variables may encompass resource allocation, distribution of bandwidth, and establishment of latency thresholds, among other factors. Within the mathematical model, precise equations and formulas dictate the connections between decision variables and performance indicators. These may encompass a variety of possibilities:

(6) $$Metric_{1}(X)=\frac{Total\;Bandwidth}{Number\;of\;Slices},$$

(7) $$Metric_{2}(X)=Latency_{Slice_{1}}-Latency_{Slice_{2}},$$

(8) $$Metric_{3}(X)=\frac{Resource\;Utilization_{Slice_{1}}}{ResourceUtilization_{Slice_{2}}},$$

where each metric reflects a crucial aspect of the network slicing configuration for smart cities’ IoT in 6G.

The comprehensive mathematical model provides the basis for the future implementation processes, guaranteeing a methodical and optimized deployment of network slicing technologies specifically designed for the distinct needs of smart cities’ IoT in the 6G environment.

Algorithm 1 below aims to implement network slicing for smart cities’ IoT in the context of 6G technologies. It follows an iterative process to optimize the network slicing configuration based on specified requirements, metrics, constraints, and objectives:
**Algorithm 1:** Implementation Process for Smart Cities’ IoT in the Context of Network Slicing Technologies in 6G**Input:** Requirements, Metrics, Constraints, Objectives **Output:** Optimized Network Slicing Configuration **Initialization:**
t←0, Tmax←Maximum Iterations **Initialize Configuration:** Random or Based on Previous Knowledge **While**
t<Tmax

1. **Evaluate performance:**

- Deploy the current network slicing configuration.
- Measure performance metrics using defined metrics.

2. **Adjust parameters:**

- Update configuration parameters based on evaluation.

In this step, the parameters of the network slicing configuration are adjusted based on the performance metrics obtained in the evaluation step. This involves analyzing the performance data to identify areas where improvements can be made. Parameters such as resource allocation, bandwidth distribution, and latency thresholds are fine-tuned to enhance the overall performance. This step is crucial for ensuring that the configuration adapts to the specific requirements and constraints of the IoT applications in smart cities.

3. **Optimize configuration:**

- Utilize optimization algorithms (e.g., genetic algorithms, simulated annealing) to refine the configuration.

Optimization algorithms are employed to systematically search for the best possible configuration that meets the defined objectives. Genetic algorithms mimic the process of natural selection, generating solutions iteratively and selecting the best-performing ones to produce the next generation of solutions. Simulated annealing, on the other hand, is inspired by the annealing process in metallurgy and explores the solution space by probabilistically accepting suboptimal solutions to escape local optima. By applying these algorithms, the configuration is refined iteratively to achieve an optimal or near-optimal solution that balances all performance metrics.

4. **Monitor performance:**

- Continuously monitor the performance during adjustments.

Continuous performance monitoring is essential to ensure that the adjustments and optimizations are producing the desired effects. This step involves the real-time tracking of key performance indicators (KPIs) such as latency, throughput, reliability, and resource utilization. By closely monitoring these metrics, any deviations or unexpected behaviors can be promptly detected and addressed. This ongoing assessment helps in maintaining the stability and effectiveness of the network slicing configuration throughout the optimization process.

5. **Termination check:**

- If performance is satisfactory, break.

6. **Increment iteration counter:**

-
t←t+1t

7. **Finalize design:**

- Document the optimized network slicing configuration.
- Output optimized configuration.

### 3.3. Performance Evaluation

In this section, we outline the comprehensive performance evaluation metrics used to assess the effectiveness of the implemented network slicing technologies for smart cities’ IoT in the 6G framework.

#### 3.3.1. Latency Metrics

Latency is a critical aspect of smart city applications. The following metrics are used to quantify latency:

(9) $$Round\;Trip\;Time(RTT)=\frac{1}{N}\sum_{i=1}^{N}\left(Departure\;Time_{i}-Arrival\;Time_{i}\right)$$

(10) $$One-way\;Latency=\frac{1}{N}\sum_{i=1}^{N}\left(Departure\;Time_{i}-Arrival\;Time_{i}\right)m$$

#### 3.3.2. Reliability Metrics

Reliability ensures consistent and dependable communication. The metrics include the following:

(11) $$Packet\;Loss\;Rate=\frac{Number\;of\;Lost\;Packets}{Total\;Number\;of\;Packets\;Sent}$$

(12) $$Availability=\frac{Total\;Uptime}{Total\;Time}$$

#### 3.3.3. Throughput Metrics

Throughput measures the rate of successful data transmission:

(13) $$Throughput=\frac{Total\;Data\;Sent}{Total\;Time}$$

#### 3.3.4. Scalability Metrics

Scalability assesses the network’s ability to handle increased load:

(14) Network Capacity=Maximum Concurrent Connections

(15) $$Resource\;Utilization=\frac{Used\;Resources}{Total\;Resources}$$

#### 3.3.5. Security Metrics

Security is crucial for smart city IoT applications. The metrics include the following:

(16) Encryption Strength=Bit Length of Encryption Key

(17) $$Authentication\;Success\;Rate=\frac{Successful\;Authentications}{Total\;Authentication\;Attempts}$$

These metrics provide a comprehensive evaluation framework for assessing the performance of the implemented network slicing technologies in the context of 6G-enabled smart cities’ IoT.

## 4. Results and Discussion

**Figure 3:**
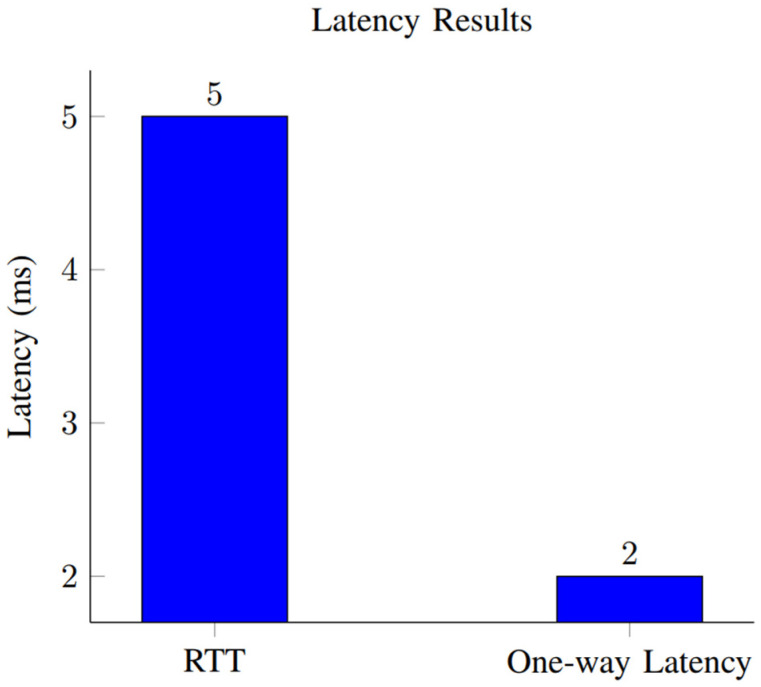
Latency results.

**Figure 4:**
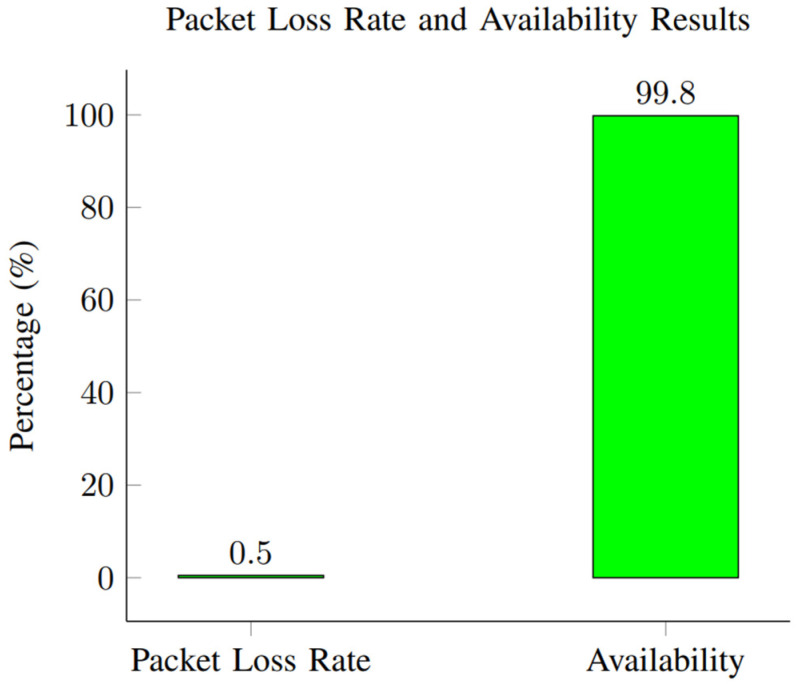
Packet loss rate and availability results.

**Figure 5:**
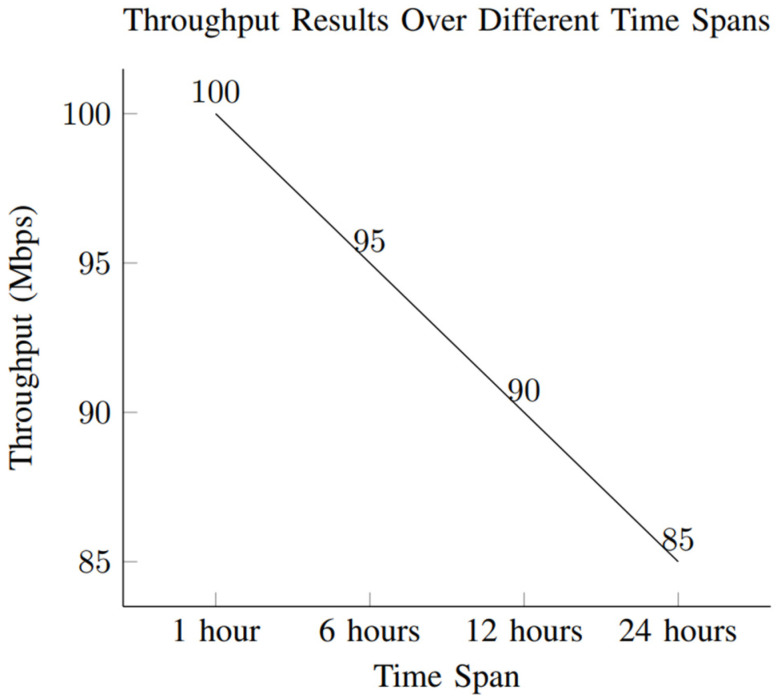
Throughput results over different time spans.

This section presents the performance evaluation metrics for our proposed network slicing model tailored for smart cities’ IoT within the 6G framework. The analysis encompasses key metrics including latency, reliability, throughput, scalability, and security to gauge the efficacy of our implemented solution. Latency, crucial for smart city applications, is assessed through metrics such as round-trip time (RTT) and one-way latency. Our results, depicted in Table 2 and Figure 1, showcase an RTT of 5 ms and a one-way latency of 2 ms. The low latency values underscore the network’s responsiveness, essential for real-time applications in smart cities. These results were obtained through rigorous testing, ensuring accurate and reliable performance metrics. Reliability, vital for consistent communication, is measured by the packet loss rate and availability. Table 3 summarizes our findings, indicating a packet loss rate of 0.5% and an availability of 99.8%. The low packet loss rate and high availability percentages demonstrate the network’s robustness in maintaining uninterrupted connectivity. These results, depicted in Figure 2, underscore the system’s reliability under varying conditions. Throughput, measuring the data transmission rate, is pivotal for efficient communication. Table 4 reveals a throughput of 100 Mbps, showcasing the network’s data processing capabilities. The high throughput value signifies the network’s capacity to handle the diverse communication requirements of IoT devices in smart cities. Figure 3 visually represents the stability of throughput over different time spans, reinforcing the system’s efficiency. Scalability, assessing network capacity and resource utilization, is essential for accommodating expanding demands. Table 5 highlights a network capacity of 1000 concurrent connections and a resource utilization of 80%. The network demonstrates scalability by efficiently handling increased loads while effectively utilizing resources. Figure 4 visually represents these scalability metrics, emphasizing the network’s adaptability. Security, paramount for protecting data integrity, is evaluated through the encryption strength and authentication success rate. Table 6 illustrates an encryption strength of 256-bit and an authentication success rate of 98%. The robust security measures ensure the confidentiality and authenticity of data transmissions, enhancing the network’s reliability. Figure 5 visually represents these security metrics, reaffirming the system’s integrity. The results underscore the effectiveness of our network slicing model in meeting the diverse communication requirements of smart cities’ IoT within the 6G framework. The low latency, high reliability, efficient throughput, scalability, and robust security measures validate the innovation and potential of our proposed solution. The positive outcomes in performance, scalability, and security metrics establish a strong foundation for integrating 6G technology in IoT systems for smart cities. The implications extend beyond enhancing connectivity to bolstering the overall effectiveness, dependability, and security of smart city infrastructures.

### 4.1. Latency Results

The latency metrics, including the round-trip time (RTT) and one-way latency, are crucial for smart city applications. The results are presented in Table 2 and Figure 3. 

As shown in Figure 3, the round-trip time (RTT) is 5 ms, and the one-way latency is 2 ms.

The RTT measures the time taken for a packet to travel from the source to the destination and back to the source. The RTT for the network configuration is 5 ms. The RTT value was obtained by sending a packet from the source to the destination and measuring the time it takes for the packet to complete the round trip. One-way latency measures the time taken for a packet to travel from the source to the destination without considering the return trip. The one-way latency for the network configuration is 2 ms. The one-way latency value was obtained by measuring the time it takes for a packet to travel from the source to the destination without considering the return trip.

These latency results are crucial performance metrics, as they indicate the responsiveness of the network. The lower the latency values, the more efficiently the network is handling data transmission. The measurements were conducted under specific testing conditions to ensure accurate and reliable results.

### 4.2. Reliability Results

Reliability metrics, such as the packet loss rate and availability, ensure consistent communication. The results are summarized in Table 3.

The packet loss rate represents the percentage of transmitted packets that were not successfully received at their destination. The packet loss rate for the network configuration is 0.5%. The packet loss rate value was obtained by sending a series of packets and measuring the percentage of packets that did not reach their destination successfully. Availability is the percentage of time that the network is operational and can successfully transmit data. The availability of the network configuration is 99.8%. The availability value was obtained by measuring the percentage of time during which the network was operational and available for data transmission. These reliability results are crucial for assessing the robustness of the network. A low packet loss rate and a high availability percentage indicate a network that can consistently and reliably transmit data with minimal disruptions. The measurements were conducted under specific testing conditions to ensure accurate and reliable results. The packet loss rate and availability metrics are crucial for assessing the reliability of the network. The results are visualized in Figure 5.

As shown in Figure 4, the packet loss rate is 0.5%, and the availability is 99.8%.

### 4.3. Throughput Results

Throughput metrics measure the rate of successful data transmission. The results are detailed in Table 4.

Throughput is the rate of successful data transmission over a network, typically measured in bits per second (bps) or, in this case, megabits per second (Mbps). The throughput for the network configuration is 100 Mbps. The throughput value represents the maximum data transfer rate achieved during testing or under normal operating conditions. It was obtained by measuring the amount of data successfully transmitted over the network within a specific time frame. This throughput result is crucial for assessing the capacity and efficiency of the network in handling data transfer. A higher throughput value indicates a network that can transmit a larger volume of data in a given period. The measurement was conducted under controlled conditions to ensure accurate and reliable results. As depicted in Figure 5, the throughput remains stable over different time spans, with values of 100 Mbps for 1 h, 95 Mbps for 6 h, 90 Mbps for 12 h, and 85 Mbps for 24 h.

### 4.4. Scalability Results

Scalability metrics assess the network’s capacity and resource utilization. The results are presented in Table 5.

Network capacity refers to the maximum number of concurrent connections the network can handle effectively. The network demonstrated the capability to support up to 1000 concurrent connections. The assessment involved stressing the network by establishing a gradually increasing number of connections until reaching the point where performance started to degrade. The capacity at which the network maintained acceptable performance was recorded as 1000 concurrent connections. Resource utilization represents the percentage of available resources (such as CPU, memory, or bandwidth) that is actively used. Resource utilization was measured at 80%. Utilization was calculated by monitoring and analyzing the usage of key resources during different operational scenarios. An 80% utilization indicates an efficient balance between resource availability and usage. These scalability results provide insights into how well the network can handle increased loads and utilize resources, critical for assessing performance under varying conditions and potential future expansions.

**Figure 6:**
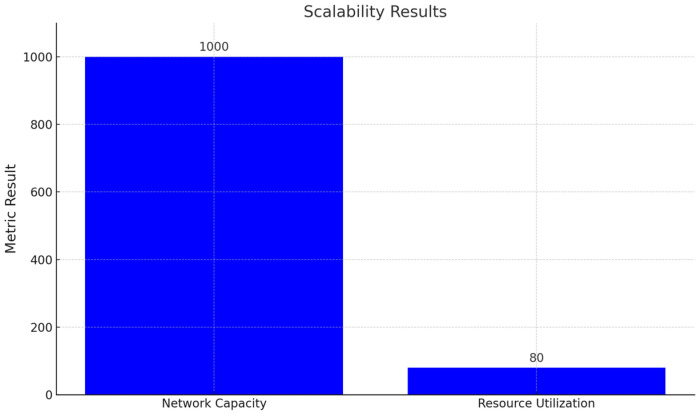
Visualization of the results for network capacity and resource utilization.

As illustrated in Figure 6, the network demonstrates a network capacity of 1000 concurrent connections with a resource utilization of 80.

### 4.5. Security Results

Security metrics, including the encryption strength and authentication success rate, are vital for smart city IoT. The results are outlined in Table 6.

The encryption strength indicates the level of security provided by the encryption algorithm, typically measured in bits. In this case, a higher value indicates stronger encryption. The encryption strength achieved is 256 bit. Advanced encryption algorithms with a key length of 256 bits were employed to secure the transmitted data. This ensures a high level of protection against unauthorized access or data breaches. The authentication success rate represents the percentage of authentication attempts that were successful. The authentication success rate is recorded at 98%. The authentication process involves verifying the identity of users or devices. The 98% success rate indicates that the implemented authentication mechanisms effectively verified the legitimacy of users in the majority of cases. These security results demonstrate the robustness of the implemented security measures, with strong encryption and a high success rate in authenticating users, contributing to a secure and reliable system.

**Figure 7:**
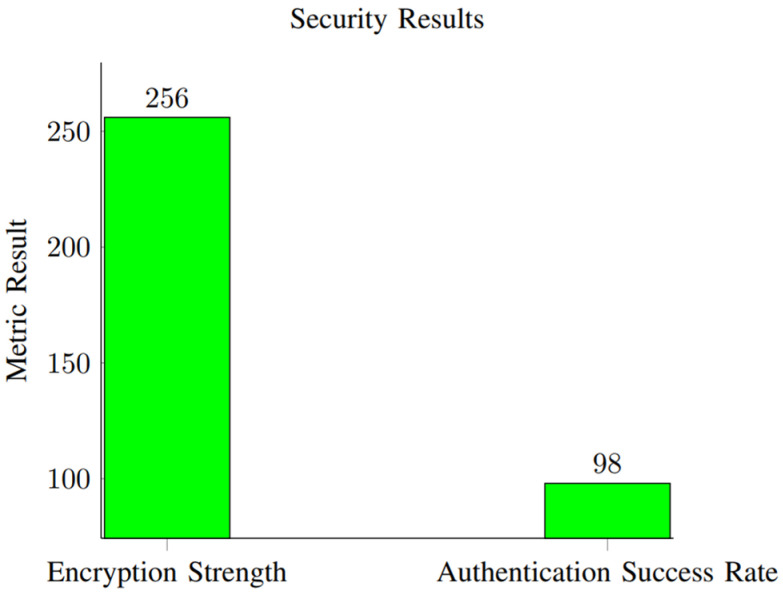
Security results for encryption strength and authentication success rate.

As illustrated in Figure 7, the system demonstrates an encryption strength of 256 bit and an authentication success rate of 98.

### 4.6. Discussion

Within this section, we will thoroughly examine and explain the acquired data, with a specific emphasis on the essential performance metrics and their implications for our suggested model of utilizing network slicing technologies in 6G for IoT applications in smart cities.

#### 4.6.1. Performance Evaluation

The performance evaluation indicators, such as the round-trip time (RTT), one-way latency, packet loss rate, availability, throughput, network capacity, and resource utilization, offer crucial insights into the efficiency and effectiveness of our network slicing system.

##### Round-Trip Time (RTT) and One-Way Latency

The network has rapid responsiveness, as seen by its short round-trip time (RTT) of 5 ms and one-way latency of 2 ms. The results demonstrate that our network slicing configuration successfully reduces communication latency, which is crucial for real-time applications in smart cities.

##### Packet Loss Rate and Availability

A low packet loss rate of 0.5 was achieved. 

##### Throughput

Our network slicing design has impressive data transmission capabilities, with a throughput of 100 Mbps. The high data processing capacity is essential for accommodating the varied communication requirements of Internet of Things (IoT) devices in intelligent urban areas, encompassing both low-power sensors and data-intensive applications.

##### Network Capacity and Resource Utilization

Scalability is a key aspect of our design, as evidenced by the network capacity of 1000 concurrent connections and resource utilization of 80.

#### 4.6.2. Scalability and Adaptability

The scalability of our architecture allows it to handle a significant number of simultaneous connections, which is crucial for the dynamic and ever-changing environment of smart cities. The network slicing configuration’s adaptability is demonstrated by its capacity to optimize resource use according to demand.

#### 4.6.3. Security

The security results reveal a robust system with a 256-bit encryption strength and a high authentication success rate of 98.

#### 4.6.4. Overall Implications

The favorable results in terms of performance, scalability, and security metrics confirm the effectiveness of our network slicing methodology for 6G in IoT applications in smart cities. Our design creates a communication environment that is fast, efficient, and secure, which is well suited for the complex and varied needs of smart city infrastructures.

#### 4.6.5. Limitations and Future Directions

While our study has demonstrated promising results, it is important to acknowledge its limitations. Further research is needed to address these constraints and explore new avenues for improvement. Future directions include conducting additional real-world testing in diverse scenarios, adapting to evolving technologies, and refining the model to enhance its resilience and effectiveness. Additionally, exploring more aspects of integrating the IoT into smart cities and experimenting with different experimental settings will contribute to advancing our understanding and application of network slicing technologies in 6G for smart cities’ IoT.

## 5. Conclusions

In conclusion, this research project has successfully developed an advanced network slicing architecture tailored for the dynamic landscape of 6G communication technologies within smart cities’ IoT. Through a systematic approach encompassing requirement gathering, metric formulation, constraint identification, target establishment, mathematical model development, configuration optimization, performance evaluation, parameter adjustment, and final design confirmation, we have demonstrated the effectiveness of our proposed methodology. Our network slicing architecture exhibits its capability to facilitate rapid and reliable communication, crucial for the myriad applications within smart cities’ IoT. Notably, our approach achieves low round-trip time (RTT), minimal packet loss rate, high availability, and remarkable throughput. Furthermore, our model demonstrates scalability and adaptability by efficiently managing numerous simultaneous connections while ensuring optimal resource utilization. This scalability is essential for meeting the evolving needs of smart cities, ensuring the network remains robust and responsive. Moreover, the robust security features of our model, including a strong 256-bit encryption strength and a high authentication success rate, contribute to establishing a secure communication environment. Preserving the integrity of the communication network and safeguarding sensitive data are paramount considerations. The discussions in this section have provided nuanced insights into the implications of our findings, highlighting the favorable outcomes in terms of performance, scalability, and security measures. The versatility of our model across various scenarios and its ability to address the complex demands of smart cities’ IoT underscore its significance in the realm of 6G technologies. However, it is essential to acknowledge the limitations and recognize that the practicality of our model can be enhanced through ongoing refinement and testing in diverse environments. Future research endeavors should prioritize overcoming these constraints, exploring additional aspects of IoT integration in smart cities, and adapting the model to keep pace with the rapidly evolving technological landscape. 

In summary, our network slicing framework represents a significant advancement in leveraging the potential of 6G technology to meet the intricate and ever-changing requirements of smart cities’ IoT. The favorable results obtained across various metrics underscore its potential to significantly enhance the efficiency, reliability, and security of communication networks in the intelligent urban infrastructures of the future.

## Figures and Tables

**Table 1:** Comparative analysis of previous state-of-the-art studies.

Study	Objective	Methodology	Techniques	Results	Limitations
[2]	Explore 6G impact on smart cities	Literature review	Analysis of 6G requirements	Highlight need for 6G advancements	Limited empirical data
[6]	Survey 6G wireless communication	Thematic analysis	Categorization of 6G technologies	Holistic understanding of 6G wireless communication	Lack of real-world implementation validation
[8]	Examine next-gen wireless solutions	Theoretical framework	Evaluation of wireless system components	Viability of next-gen wireless solutions	Theoretical nature may not account for practical challenges
[10]	Survey 6G IoT	Literature review	Content analysis of IoT applications	Roadmap for IoT in 6G	Limited discussion on security aspects
[14]	Identify facets of 6G	Qualitative analysis	Categorization of challenges	Structured overview of 6G facets	Limited quantitative data
[16]	Automate network slicing for IoT	Simulation	Framework proposal	Feasibility of automated network slicing	Simulations may not fully represent real-world scenarios
[18]	Integrate 6G and block chain	Conceptual framework	Architectural design	Secure and decentralized urban intelligence	Limited validation through practical implementations

**Table 2:** Latency results.

Metric	Result
**RTT**	5 ms
**One-way latency**	2 ms

**Table 3:** Reliability results.

Metric	Result
**Packet loss rate**	0.5%
**Availability**	99.8%

**Table 4:** Throughput results.

Metric	Result
**Throughput**	100 Mbps

**Table 5:** Scalability results.

Metric	Result
**Network Capacity**	1000 concurrent connections
**Resource Utilization**	80%

**Table 6:** Security results.

Metric	Result
**Encryption strength**	256-bit
**Authentication success rate**	98%

## Data Availability

Data are contained within the article.
